# Microbial diversity of milk ghee in southern Gansu and its effect on the formation of ghee flavor compounds

**DOI:** 10.1515/biol-2022-0516

**Published:** 2022-12-12

**Authors:** Zewen Liu, Huixia Li, Dandan Gao, Junhong Su, Yuxin Su, Zhongren Ma, Zhiqiang Li, Yanjiao Qi, Gongtao Ding

**Affiliations:** Life Science and Engineering College, China-Malaysia National Joint Laboratory, Biomedical Research Center, Northwest Minzu University, Lanzhou, 730030, PR China; Ganan Research Institute of Yak Milk, Hezuo, 747000, PR China; China-Malaysia National Joint Laboratory, Biomedical Research Center, Northwest Minzu University, Lanzhou, 730030, PR China; Key Laboratory of Biotechnology and Bioengineering of State Ethnic Affairs Commission, Biomedical Research Center, Northwest Minzu University, Lanzhou, 730030, PR China; Department of Gastroenterology and Hepatology, Erasmus MC-University Medical Center, Rotterdam, Netherlands; Department of Dentistry, Key Laboratory of Oral Diseases of Gansu Province, Key Laboratory of Stomatology of State Ethnic Affairs Commission, Northwest Minzu University, Lanzhou, 730030, PR China; Key laboratory for utility of environment-friendly composite materials and biomass in universities of Gansu Province, Lanzhou, 730030, PR China

**Keywords:** yak ghee, lactic acid bacteria, flavor substances, metabolomics

## Abstract

Ghee is a traditional Tibetan dairy product with high-fat content, low yield, plasticity, caseation, and rich nutrition. In this study, we analyzed the diversity of microbial communities in yak milk and ghee samples at high and low altitudes, especially the *Lactobacillus* genus, and further used metabolomic techniques to compare the differences in metabolites in yak ghee at different altitudes. The results showed that the increase in altitude had a significant and generally inhibitory effect on the microbial community diversity in milk ghee, and yak milk at high altitude was abundant in nutrients, which could antagonize the negative impact of increased altitude. Using non-targeted metabolomics, we infer the composition of flavor compounds in ghee: nine kinds of carboxylic acids, 11 kinds of esters, six kinds of ketones, two kinds of alcohols, and four kinds of alkene compounds, among which the key flavor compounds are dl-2-(acetylamino)-3-phenylephrine acid, 1-(4-methoxyphenyl)-2-propanone, sebacic acid, Lysope 18:1, and uracil 1-beta-d-arabinofuranoside. These flavor substances are found in *Lactococcus*, *Lactobacillus*, and *Streptococcus*. With the participation of *Lactobacillus*, it is synthesized through biosynthesis of alkaloids derived from ornithine, lysine, and nicotine acid and glyoxylate and decarboxylate metabolism, among which *Lactococcus* plays a key role. In this study, a variety of lactic acid bacteria related to ghee fermentation were screened out, revealing the composition of volatile flavor compounds in Gannan yak milk ghee in the Qinghai–Tibet Plateau and providing a reference for further key volatile flavor compounds and the formation mechanism of flavor compounds.

## 1 Introduction

Ghee is a traditional Tibetan dairy product made from yak milk through a series of fermentation, heat clarification, and drying. Yaks belong to the Bovine subfamily and the *Bos grunniens* classification and are mainly distributed in the Himalayas of Nepal, the Kashmir Plateau in India, Tibet, Mongolia, and Bhutan [1]. The main components of yak milk are fat (6–10%), protein (5.5%), lactose (5%), and minerals (1.1%) [2]. The nutritional value of yak milk is increasingly acknowledged and used in products such as cheese, cream tea, and yogurt. Traditional Tibetan ghee has important culinary uses such as seasoning, cooking, frying, and even due to its beautiful appearance, granular and semi-solid form, pleasant smell, excellent taste, and a high proportion of polyunsaturated fatty acids [3] it is used in religious ceremonies.

Yaks in Gansu Province are widely distributed in 22 pastoral and semi-agricultural and semi-pastoral areas in Gannan Tibetan Autonomous Prefecture and the foothills of the Qilian Mountains. Gannan Tibetan Autonomous Prefecture has 1.13 million yaks, making up 78% of the total number of yaks in Gansu. Gannan yak is mainly distributed in Hezhou City, Maqu, Luque, Xiahe, Zhuoni County, and Diebu County. Yak is a unique native breed and animal husbandry resource. It belongs to herbivorous ruminant livestock and is recognized as the “boat on the plateau.”

The traditional production model of fermented dairy products has been retained and continued in many regions at home and abroad. Lactic acid bacteria, which play a vital role, have always been the focus of domestic and foreign researchers. The microbial composition of fermented dairy products has been investigated since the end of the nineteenth century [4]. In 2001, Beukes et al. [5] studied the microbial diversity in traditional fermented milk in South Africa. She identified the selected strains by isolation and culture method by physiological, biochemical, and API50CH reagent strips. In 2006, Meulen et al. [6] conducted a series of studies on the diversity of lactic acid bacteria in Romanian traditional fermented milk and identified the isolated strains by rep-PCR fingerprinting technology and 16srRNA gene sequencing. In recent years, Chinese scholars have also studied the diversity of lactic acid bacteria in fermented dairy products from diverse regions. In 2007, Zhengzhou University and the National Institute of Livestock and Grassland of Japan isolated and identified the microorganisms in the customary Tibetan fermented dairy product Qual. In 2008, Watanabe et al. used pure culture and molecular biology methods to systematically explore lactic acid bacteria’s diversity and community structure in naturally fermented dairy products in Mongolia. In 2008, Zhang et al. [7] studied the chemical components and microorganisms of naturally fermented yak milk yogurt in Qinghai, China, and found that the content of lactic acid bacteria was 9.18 ± 0.8151.

Many studies since 2010 have shown that the probiotic effects of lactic acid bacteria derived from fermented dairy products on the human body have attracted more and more attention from researchers. Clinical trials have confirmed that daily consumption of lactic acid bacteria products potentially affects various diseases [8]. It has been noted that probiotic lactic acid bacteria can slow down irritable bowel syndrome [9] and relieve neonatal colic [10]. *Lactobacillus*, especially *Lactobacilli*, has probiotic properties such as cholesterol-lowering, diabetes treatment, bacteriostasis, anticancer, and stimulation of immune response [3,11–13]. Numerous studies have also reported the contribution of lactic acid bacteria from metabolites to food formation during dairy fermentation. Production of bacteriocin by lactic acid bacteria plays an important role in effectively preventing the spoilage of fermented food and the infection of pathogenic bacteria [14–17]. *Lactobacillus exopolysaccharides* have also received extensive attention in human health, mainly including improving the quality of fermented milk [5,18,19], anti-tumor effects [20], regulating host immune responses, and antioxidative response [21].

Metabolomics is a new discipline that simultaneously conducts a qualitative and quantitative analysis of all small molecule metabolites of a certain organism or cell in a specific physiological period. The emerging omics technology has also become an important part of systems biology. In this study, combined with a high-throughput sequencing platform and bioinformatics analysis methods, yak milk ghee samples from herdsmen families in the southeastern Qinghai–Tibet Plateau and Gannan Plateau, from 2,800 to 4,500 m altitude, were used for bacterial amplicon sequencing. This study revealed the impact of differential changes in the microbial community of yak milk ghee at different altitudes in a high altitude environment from the genome level. At the same time, GC-MS (headspace solid-phase microextraction) technology was used for yak milk ghee samples to compare the differences in metabolites in yak milk ghee samples at different altitudes from the metabolome level, to explore the key flavor substances, and to reveal the Qinghai–Tibet Plateau in China. The composition of volatile flavor compounds in Gannan yak milk ghee provides a reference for further strategic explosive flavor compounds and the formation mechanism of flavor compounds.

## 2 Materials and methods

### 2.1 Sample collection

The sampling site of this experiment is the Gannan Plateau in the southeastern Qinghai–Tibet Plateau, with altitudes ranging from 2,800 to 4,500 m, and 15 milk samples and 15 corresponding naturally fermented milk ghee samples from about 15 herdsmen families were collected. The milk ghee samples were quickly stored in a −80°C refrigerator, and all samples were subjected to 16 s high-throughput sequencing and metabolomics determination by Beijing Nevogene Technology Co., Ltd. Among them, the yak milk and ghee in high altitude areas are abbreviated as YM and YG, respectively, and the sample numbers are 1–12. YM and YG sampling was conducted in mid-July 2021 in Bola Township (E102.824425, N34.867262, altitude was 3,189 m), Xiahe County, Gansu Province; the Sumatran milk and the corresponding fermented ghee in low altitude areas are abbreviated as SM and SG, respectively, and the sample numbers are 1–12. Sampling of SM and SG was conducted in mid-July 2021 in Shuangquan Village (E103.581858, N35.475211, altitude was 2,064 m), Guanghe County, Gansu Province.

### 2.2 DNA extraction and sequencing

The CTAB/SDS method extracted total genomic DNA from the samples. Monitor DNA concentration and purity on a 1% agarose gel and dilute DNA to 1 µg/µL using sterile water according to attention. Amplify 16S rRNA/18S rRNA/ITS genes in different regions (16S V4/16S V3/16S V3-V4/16S V4-V5, 18S V4/18S V9, ITS1/ITS2, Arc V4) using specific primers. All PCR reactions were performed using 15 µL of Phusion® High-Fidelity PCR Master Mix (New England Biolabs); 0.2 µM forward and reverse primers and approximately 10 ng of template DNA. Thermal cycling consisted of initial denaturation at 98°C for 1 min, followed by 30 cycles of denaturation at 98°C for 10 s, annealing at 50°C for 30 s, extension at 72°C for 30 s, and a final extension at 72°C for 5 min [22].

Sequencing libraries were generated using the TruSeq^®^ DNA PCR-Free Sample Preparation Kit (Illumina, USA) following the manufacturer’s recommendations and added index codes. Library quality was assessed on a Qubit@2.0 fluorometer (Thermo Scientific) and an Agilent Bioanalyzer 2100 system. Finally, the library was sequenced on the Illumina NovaSeq platform, and 250 bp paired-end reads were generated.

### 2.3 Metabolite extraction and analysis

Take 100 µL of the sample and put it in an EP tube, add 400 µL of 80% methanol aqueous solution, vortex, let stand in an ice bath for 5 min, and centrifuge at 15,000*g* for 20 min at 4°C; take a certain amount of supernatant and add mass spectrometry-grade water to dilute the methanol content to 53%; centrifuged at 15,000*g*, 4°C for 20 min, collected the supernatant, and injected into LC-MS for analysis.

UHPLC-MS/MS analyses were performed using a Vanquish UHPLC system (Thermo Fisher, Germany) coupled with an Orbitrap Q Exactive™ HF mass spectrometer (Thermo Fisher, Germany) in Novogene Co., Ltd (Beijing, China).

Samples were injected onto a hypersil gold column (100 mm × 2.1 mm, 1.9 µm) using a 17 min linear gradient at a flow rate of 0.2 mL/min. The eluents for the positive polarity mode were eluent A (0.1% FA in water) and eluent B (methanol). The eluents for the negative polarity mode were eluent A (5 mM ammonium acetate, pH 9.0) and eluent B (methanol). The solvent gradient was set as follows: 2% B, 1.5 min; 2–85% B, 3 min; 100% B, 10 min; 100–2% B, 10.1 min; 2% B, 12 min. Q Exactive™ HF mass spectrometer was operated in positive/negative polarity mode with spray voltage of 3.5 kV, capillary temperature of 320°C, sheath gas flow rate of 35 psi and aux gas flow rate of 10 L/min, S-lens RF level of 60, Aux gas heater temperature of 350°C [23,24].

### 2.4 Data statistics and analysis

R language was utilized to produce relative abundance shock bar charts to describe community species composition and species abundance information. Beta diversity analysis was performed using QIIME software. Then a tree-like structure was constructed using the weighted Bray–Curtis calculation method to present the degree of similarity or difference in community composition in different samples.

The identified metabolites were annotated using the KEGG database, the HMDB database, and the LIPID Maps database. In the multivariate statistical analysis part, the data were transformed using metaX data processing software. Then principal component analysis (PCA) and partial least squares discriminant analysis were performed to obtain the VIP value of each metabolite. In the univariate analysis part, the statistical significance (*p*-value) of each metabolite between the two groups was calculated based on the *t*-test, and the fold change (fold change) of the metabolite between the two groups, i.e., the FC value was calculated. Correlation analysis between the differential metabolites (Pearson correlation coefficient) was performed using R language cor(), and the statistical significance was achieved by cor. M test () in R language. *p*-value <0.05 was considered statistically significant, and R language was used in the complot package in Plotting Correlation Plots. Bubble plots were drawn with the R package ggplot2, the KEGG database was used to study the functions and metabolic pathways of metabolites, and other statistical analyses and mapping were done by Origin 2020.

## 3 Results and discussion

### 3.1 Microbial community richness and species composition

**Figure 1:**
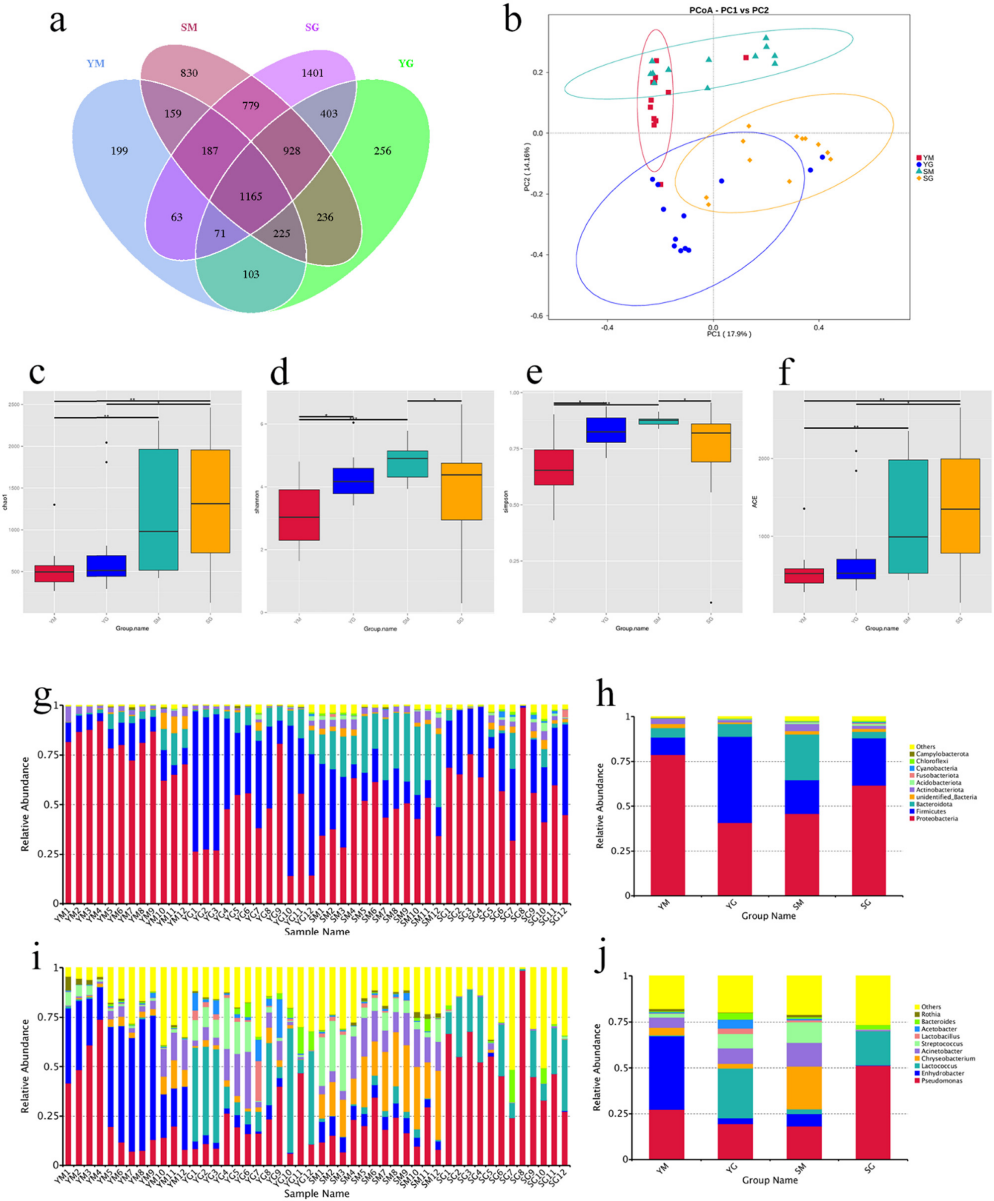
(a) Venn diagram of microbial community OTUs in milk and ghee samples at high and low altitudes. (b) Principal co-ordinates analysis (PCoA) analysis of microbial community diversity. Microbial diversity alpha analysis: (c) Chao1 index, (d) Shannon index, (e) Simpson index, (f) ACE index. (g and h) Histograms of the sample community structure at the phylum level. (i and j) Histograms of the sample community structure at the genus level (YM, YG, SM, and SG refer to milk and ghee at high and low altitudes, respectively. *Indicates a significant difference at the *p* < 0.05 level, ** indicates a significant difference at the *p* < 0.01 level.).

The operational taxonomic unit (OTU) cluster analysis and Venn diagram showed that the OTU numbers of the four groups of samples were 2,172 in the YM group, 3,387 in the YG group, 4,509 in the SM group, and 4,997 in the SG group, of which the YM and the YG group shared 1,564 OTUs, YM and SM group share 1,736 OUTs, and YG and SG share 2,567 OUTs (Figure 1a). From this, we can draw the following conclusions [1]: the richness of microbial communities in milk and ghee samples from high altitude areas is lower than that of corresponding samples from low altitude areas [2]; whether, in high altitude or low altitude areas, the richness of the microbial community increased during the fermentation process known as ghee, more pronounced at higher altitudes. It is speculated that the reason for this is that the temperature in the high altitude areas of the Qinghai–Tibet Plateau is significantly lower than that in the low altitude areas, and the low temperature is not conducive for the colonization and reproduction of microorganisms. Hence, the community richness is low [25]. In the process of milk ghee fermentation, many bacteria such as lactic acid bacteria, are involved, and the microorganisms involved in the fermentation process are the newly added OTU units, which are consistent with the results of the study and are convincing.

The top ten bacterial phyla in milk and ghee collected from high and low altitude areas are shown in Figure 1g and h. The main microbial phyla in milk and ghee samples are *Proteobacteria* (40.8–78.7%), *Firmicutes* (9.7–48.1%), and *Bacteroidota* (3.5–25.6%), the total proportion of the three bacterial phyla exceeded 95%. With the increase in altitude, the relative abundances of *Proteobacteria*, *Firmicutes*, and *Bacteroidota* in the samples all changed significantly, which indicates that the microbial community structure is greatly affected by the altitude. In the process of milk fermentation, *Firmicutes* in both high and low altitude areas increased significantly, and their relative proportions increased by 38.4 and 7.51%, respectively. The *Bacteroidota* in milk ghee in high altitude areas was lower than that in low altitude areas (40.8 vs 61.7%), while *Firmicutes* were higher than at lower altitudes (48.1 vs 26.2%).

The top ten genera of bacteria in milk and ghee collected from high and low altitude areas are shown in Figure 1i and j. The main microbial genera in milk and ghee samples are *Pseudomonas* (18.1–51.4%), *Enhydrobacter* (3.1–40.1%), *Lactococcus* (2.6–27.3%), and *Chryseobacterium* (2.5–23.3%). Among them, the relative abundances of *Enhydrobacter*, *Lactococcus*, and *Chryseobacterium* changed significantly with the increase in altitude. With the altitude getting higher, *Enhydrobacter* in milk increased from 6.8 to 40.1%, but *Chryseobacterium* decreased from 23.4 to 4.3%. The relative abundance of *Pseudomonas* in ghee decreased from 51.4 to 19.4% with altitude. By further comparing the bacterial species in different samples, we can find that the species with higher abundance include *Moraxella osloensis*, *Lactococcus lactis*, *Chryseobacterium anthropi*, and different kinds of ghee samples have their unique dominant strains, such as *M. osloensis* is 40.1% abundant in YM samples but *L. lactis* is the most in YG samples (23.2%). This shows that the main bacterial species of milk have changed during the fermentation process.

Lactic acid bacteria are a general term for a class of bacteria that can produce large amounts of lactic acid from fermentable carbohydrates, and lactic acid bacteria genera common in naturally fermented dairy products consist of *Lactobacillus*, *Bifidobacterium*, *Leuconastoc*, *Pediococcus*, *Streptococcus*, *Enterococcus*, and *Lactococcus*. The most commonly used commercial promoters are *Lactococcus*, *Lactobacillus*, *Streptococcus*, and *Bifidobacterium*. *Lactococcus*, *Streptococcus*, and *Lactobacillus* were detected in this study, all of which are commonly used commercially. Among them, the three lactic acid bacteria genera (*Lactococcus*, *Streptococcus*, and *Lactobacillus*) in the milk samples from the high altitude area are all lower than those in the low altitude area, and the temperature drop caused by the increase in altitude is still considered. Still, in the ghee sample, the three genera in the high altitude area are all higher than those in the low altitude area. It is speculated that the reason for this is that the content of various nutrients such as protein and fat in high altitude yak milk is higher than that of ordinary milk, and so it is more conducive for the reproduction and fermentation process of lactic acid bacteria [26]. The content of corresponding bacteria in the complementary ghee products is also higher, which also provides a basis for the higher nutritional value of high altitude milk ghee. During the fermentation process of high altitude yak milk, the relative abundance of the three lactic acid bacteria increased significantly. *Lactococcus* has the largest growth rate, indicating that it is the dominant bacteria genus and plays a key role in fermentation. *Lactococcus* in low altitude areas increased significantly, but *Streptococcus* and *Lactobacillus* decreased significantly. It is speculated that the reason may be the lack of nutrients in low altitude milk or fermentation. The acidic substances and metabolic wastes produced in the process inhibit the reproduction of these two genera [27]. In conclusion, the elevation of yak milk has a significant and generally inhibitory effect on the microbial community structure in milk ghee. The high altitude yak milk is rich in nutrients. *Lactococcus*, *Lactobacillus*, and *Streptococcus* are involved in the fermentation process, and *Lactococcus* plays a key role.

### 3.2 Microbial community diversity and difference

Alpha diversity (α diversity) refers to the diversity within a specific area or ecosystem and is a comprehensive indicator of richness and evenness. Alpha diversity is mainly related to two factors: one is the number of species, i.e., richness; the other is diversity, the uniformity of individual distribution in the community [28]. Alpha diversity analysis was performed on milk and ghee samples collected from high and low altitude areas, and the results are shown in Figure 1. From the altitude perspective, the α diversity of milk and ghee decreased to varying degrees with the increase in altitude. For example, Chao1 index: SM 1237.78, YM 533.49; SG 1328.59, YG 749.63 (Figure 1c); Shannon index: SM 4.792, YM 3.19; SG 3.949, YG 4.298 (Figure 1d). The Simpson and ACE indices also decreased (Figure 1e and f), which indicated that with the increase in altitude, the species richness of the microbial community in both milk and fermented ghee decreased. The community structure evolved from complex to single, which is the result of the combined effect of temperature reduction and vegetation type reduction caused by the increase in altitude [29]. This result is consistent with the results of the study [30], indicating that the experimental data are credible. From the perspective of fermentation, during the process of fermenting milk into ghee, the four diversity indices of samples from high altitude areas all increased, indicating that the richness and complexity of the samples increased; Chao1 and ACE indices of samples from low altitude areas increased, while the Shannon and Simpson indices decreased (Figure 1c–f), the corresponding significant *p*-values are reflected in the graph.

β-Diversity, also known as inter-habitat diversity, refers to the dissimilarity of species composition among different habitat communities along with the environmental gradient or the replacement rate of species along the environmental angle. The main ecological factors controlling β-diversity are soil, landform, and disturbance [31]. Based on Bray Curtis, PCoA and similarity analysis were used to compare the differences in community species composition between milk and ghee samples in high and low altitude areas. The closer the sample points are, the more similar the community structure is. The first and second principal coordinates explained 17.9 and 14.1% of the variation in the microbial community composition of the two samples, respectively (Figure 1b). The results of PCoA analysis showed that the aggregation positions of YM and GM and the YG and SG group in the two groups of ghee samples overlapped to a certain extent, indicating that the change of altitude caused a certain difference but the effect was not large. However, YM, YG, SM, and SG have obvious changes in the aggregation positions of the milk ghee samples before and after fermentation, which indicates that during the fermentation process, the microbial communities in the milk ghee in the high and low altitude areas have changed significantly. The difference is significant (*p* < 0.01).

### 3.3 LEfSe analysis and community function prediction

**Figure 2:**
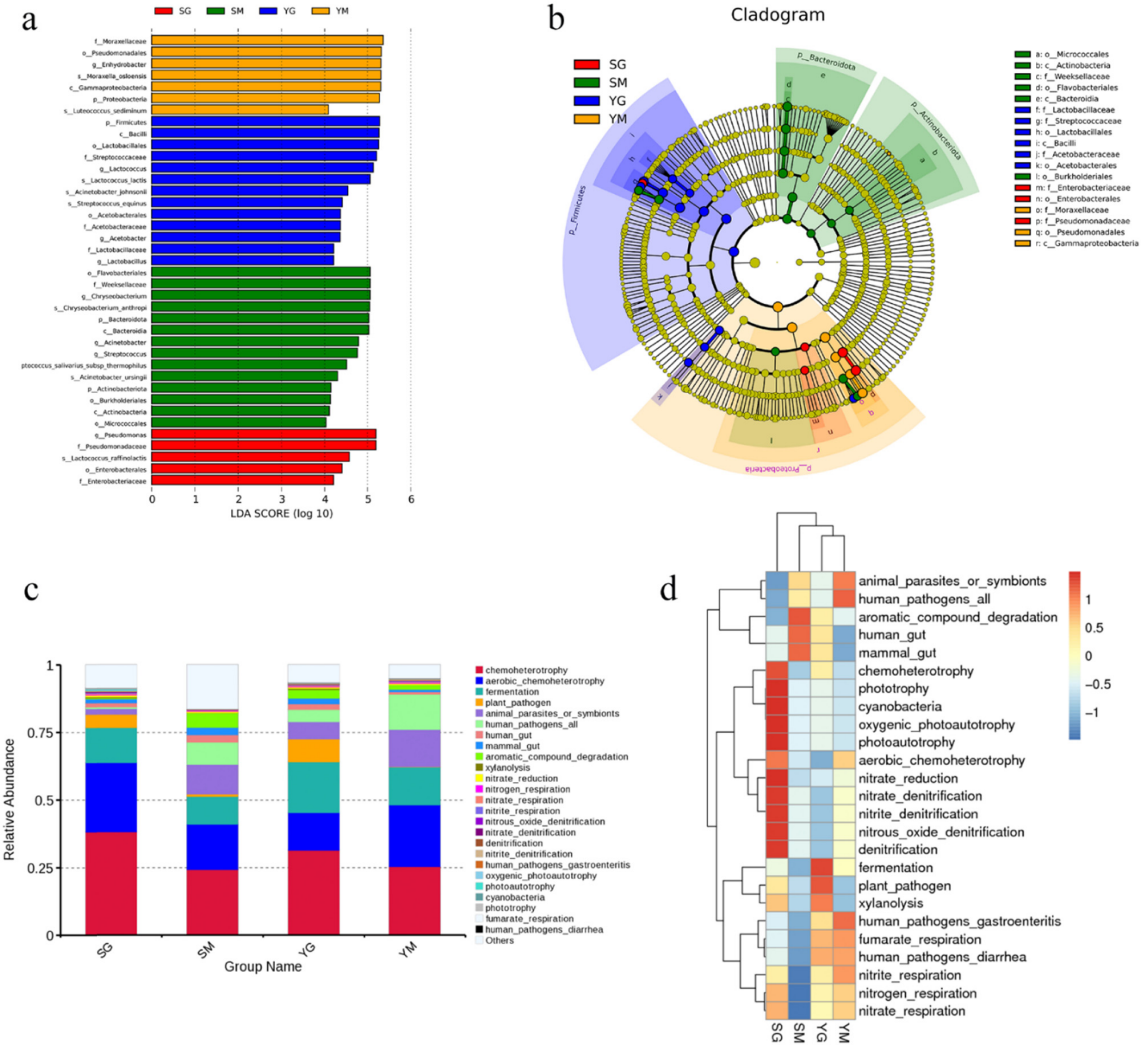
(a) Score map of different species. (b) Annotated branching map of other species. (c) FAPROTAX functional annotation relative abundance column chart. (d) FAPROTAX feature annotation clustering heatmap.

LEfSe analysis is an analysis tool for discovering and interpreting high-dimensional data related to genes, pathways, taxa, and other biomarkers. In microbial diversity analysis, LEfSe can analyze differences between groups of microorganisms, such as differences in flora etc. [32]. The results of the analysis of the contribution of different species to the difference are shown in Figure 2a (LDA score >4). There were seven abundant bacterial clades in the high altitude YM group, 13 in the YG group, and 14 and 5 in the low altitude SM and SG groups, respectively. In the process of fermenting milk into ghee, compared with the YM group, the fermentation-related lactic acid bacteria in the YG group are *Lactobacillales* (order), *Streptococcaceae* (family), *Lactococcus* (genus), *L. lactis* (species), *Streptococcus equinus* (species), *Lactobacillaceae* (family), *Lactobacillus* (genus), a total of seven types. These seven types of lactic acid bacteria play an important role in the fermentation process and are closely related to the formation of flavor substances. Similarly, comparing the SM group, the SG group that caused the difference with fermentation-related lactic acid bacteria are *Lactococcus raffinolactis* (species), *Enterobacterales* (order), *Enterobacteriaceae* (family), a total of three types; these three types of lactic acid bacteria play an important role in the fermentation process. Similarly, we can find that the ghee samples at high altitudes and low altitudes have many types of microorganisms that play a role in fermentation, indicating that the nutritional value of yak milk ghee at high altitudes is higher. These lactic acid bacteria need to be developed and utilized. Species with significant differences in all-milk ghee samples were annotated and analyzed (Figure 2b).

In the analysis of microbial diversity, the FAPROTAX database is a commonly used community function annotation database. The FAPROTAX database has many applications and good results in annotating the functions of soil microorganisms in marine lake biochemical processes (sulfur, nitrogen, hydrogen, and carbon cycles) [33]. Community function annotation was performed on the microbial communities in milk and ghee samples at high and low altitudes using the FAPROTAX database. The results are shown in Figure 2c. The sample microbial communities mainly include the following functions: chemoheterotrophy, aerobic chemoheterotrophy, fermentation, plant pathogen, animal parasites or symbionts, and human pathogens all. There are differences in the specific proportions. Here we focus on the analysis of the fermentation function. The number of microorganisms that perform fermentation functions in milk and ghee in high altitude areas is more than that in low altitude areas, whether it is ghee or unfermented milk. In the fermentation process, YG compared with the YM group and the SG compared with the SM group, the number of microorganisms performing fermentation function was significantly increased (*p* < 0.05). Therefore, a unique flavor was also formed, which is consistent with the results of Zhang et al. [7]. The heat map of FAPROTAX analysis further revealed the differences in microbial community function between the samples at different altitudes (Figure 2d).

### 3.4 Metabolome differences between yak milk and ghee

**Figure 3:**
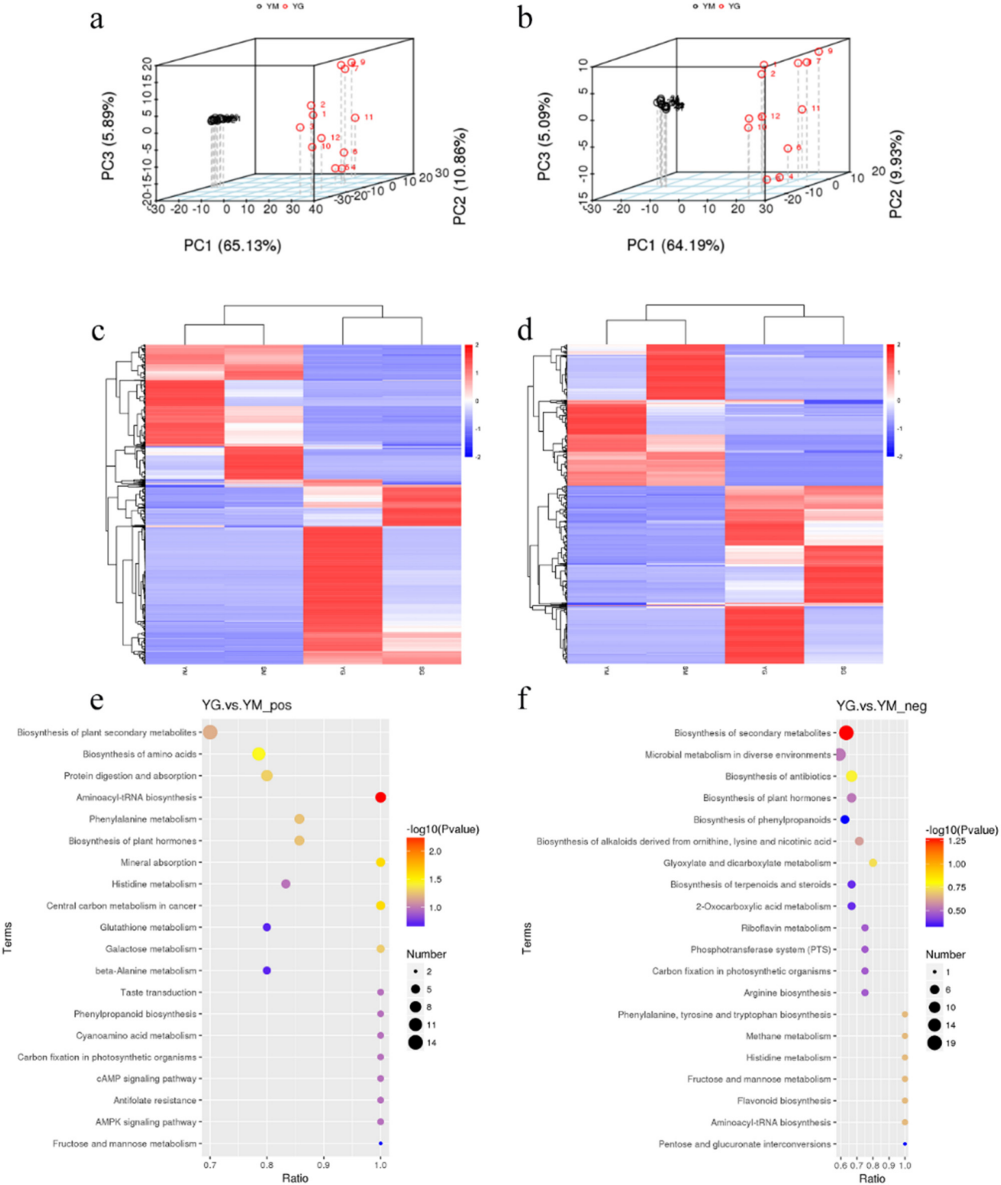
Three-dimensional structure of PCA plots based on metabolomes under positive mode (a) and negative mode (b) of yak milk and ghee samples at high altitude (YM vs YG). Hierarchical clustering based on differential metabolites under positive mode (c) and negative mode (d) of yak milk and ghee samples at high and low altitudes. Metabolomic pathway enrichment is based on metabolomes under positive mode (e) and negative mode (f) of yak milk and ghee samples at high altitudes (YM vs YG).

To explore the composition and formation process of flavor compounds in Gannan yak milk ghee, we performed a non-targeted metabolomic analysis on the collected samples. The results are shown in Figure 3. In this metabolomic analysis, quality control is highly consistent in positive or negative mode, ensuring accuracy and reproducibility [34]. Based on the identified metabolites, the PCA plots deciphered the similarity and variability between the metabolites of the samples (Figure 3a and b), and it was found that the metabolites of milk and ghee were significantly different (*p* < 0.01), and the ghee formed after fermentation.

Hierarchical clustering further proved that altitude had a significant effect on the types of metabolites in milk and ghee (*p* < 0.05), but the metabolite differences were much lower than those in milk and ghee before and after fermentation (*p* < 0.01; YM vs YG, SM vs SG). The positive and negative patterns yielded consistent conclusions (Figure 3c and d).

Comparing the differences in the types of specific metabolites between the YM group and the YG group, we can speculate the composition of flavor substances in the milk ghee (see the report for particular metabolites), and there are significant differences between the yak milk and the fermented ghee samples and ghee contains some unique substances. There are nine kinds of carboxylic acids, 11 kinds of esters, six kinds of ketones, two kinds of alcohols, four kinds of alkene compounds, one kind of aniline compounds, and one kind of phenol compounds, among which the substances with higher content are caproic acid, octanoic acid, 4-methyl base-phenol, and *n*-decanoic acid. Our results are roughly the same as those determined by Dorea et al. [35] using GC/MS combined technique, indicating that the inference is reasonable.

To explore the formation process of flavor compounds in milk ghee, we performed a metabolomic pathway enrichment comparative analysis between the YM group and the YG group. The results are shown in Figure 3e and f. During the fermentation process of milk, the metabolic pathways changed significantly (*p* < 0.05) the processes like biosynthesis of amino acids, aminoacyl-tRNA biosynthesis, biosynthesis of alkaloids derived from ornithine, lysine, and nicotinic acid, glyoxylate and dicarboxylate metabolism. Most of these are related to the synthesis or metabolism of the flavor of above-mentioned substances, such as the synthesis of carboxylic acids and the synthesis of esters, indicating that they are all part of the fermentation process of yak milk, which provides the basis for the commercial synthesis of milk ghee at the molecular level and mechanism.

### 3.5 Correlation analysis of metabolome and microbiome changes

**Figure 4:**
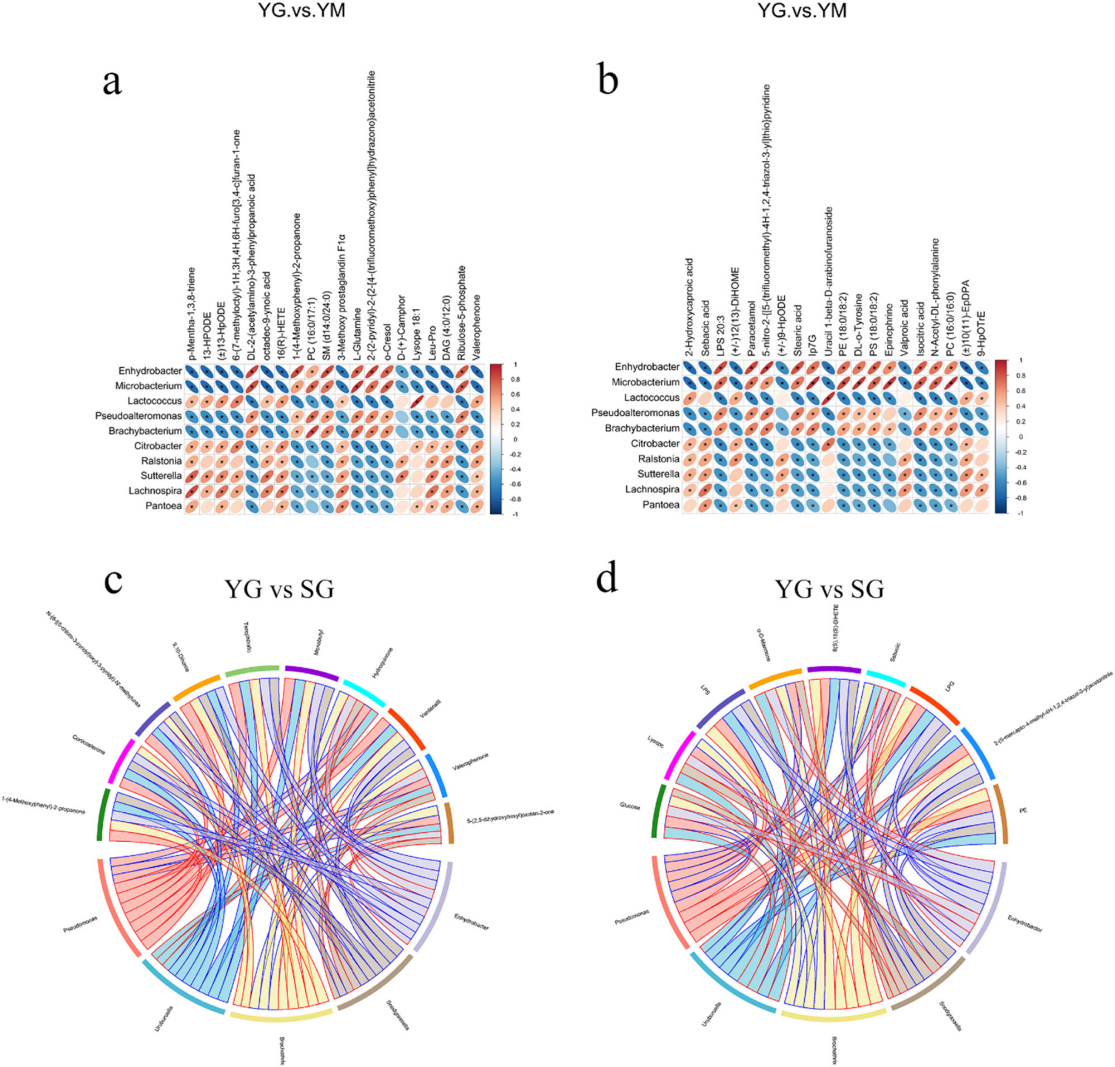
Correlation heatmap analysis based on genera and metabolomes under positive mode (a) and negative mode (b) of yak milk and ghee samples at high altitude (YM vs YG). Correlation chord diagram analysis based on genera and metabolomes under positive mode (c) and negative mode (d) of ghee samples at high and low altitudes (YG vs SG).

To explore the correlation between metabolome and microbiome changes, we compared four groups of samples of milk and ghee in high and low altitude areas and selected the ten bacterial genera and 20 metabolites with the greatest differences in each group. The results of the correlation analysis are shown in Figure 4. The YM and YG groups were compared to explore the lactic acid bacteria and their metabolites that played a key role in the fermentation process (Figure 4a and b). The results showed that the lactic acid bacteria in the fermentation process included *Enhydrobacter* and *Lactococcus*, of which dl-2-(acetylamino)-3-phenylpropanoid acid, 1-(4-methoxyphenyl)-2-propanone, SM (d14:0/24:0), 2-(2-pyridyl)-2-{2-[4-(trifluoromethoxy)phenyl]hydrazono}acetone, and paracetamol were significantly positively correlated with *Enhydrobacter* (*r* > 0.90); Lysope 18:1 and uracil 1-beta-d-arabinofuranoside were significantly positively correlated with *Lactococcus* (*r* > 0.9). So it can be speculated that the two lactic acid bacteria in the fermentation process mainly produced seven metabolites, which are the source of flavor substances.

At the same time, to explore the effect of altitude on metabolites in ghee, we compared the SM and the SG group. We correlated their metabolome and microbiome differences (Figure 4c and d). The only difference is *Enhydrobacter*, and the first five compounds with significant differences are PE (4:0/20:4), 2-(5-mercapto-4-methyl-4*H*-1,2,4-triazole-3-yl) acetonitrile, LPG 16:1, sebacic acid, and 8(*S*), 15(*S*)-DiHETE, of which *Enhydrobacter* and 1,5,8-trihydroxy-9-oxo-9*H*-xanthan-3-yl beta-d-glucopyranoside were significantly positively correlated (*r* > 0.9).

## 4 Conclusion

In this study, we analyzed the diversity of microbial communities in milk and ghee samples at high and low altitudes, especially the variety of lactic acid bacteria, and further used metabolomics to compare the differences in metabolites in milk and ghee at different altitudes. The study showed that the increase in altitude had a significant and generally inhibitory effect on the microbial community structure in milk ghee, and yak milk at high altitude was rich in nutrients, which could antagonize the negative impact of increased altitude. By comparing the differences of lactic acid bacteria in yak milk and ghee samples before and after fermentation, we screened out three lactic acid bacteria genera, *Lactococcus*, *Lactobacillus*, and *Streptococcus*, among which *Lactococcus* played a key role. Comparing the differences in the metabolites in yak milk ghee samples at different altitudes at the metabolome level, we can infer the composition of flavor substances in ghee as nine carboxylic acids, 11 esters, six ketones, two alcohols, four alkene compounds, etc., among which the key flavor substances are dl-2-(acetylamino)-3-phenylpropanoid acid, 1-(4-methoxyphenyl)-2-propanone, sebacic acid, Lysope 18:1 and uracil 1-beta-d-arabinofuranoside. The process of their synthesis mainly includes biosynthesis of amino acids, aminoacyl-tRNA biosynthesis, biosynthesis of alkaloids derived from ornithine, lysine, and nicotinic acid, and glyoxylate and dicarboxylate metabolism. In this study, several lactic acid bacteria related to ghee fermentation were screened out, and the composition of volatile flavor compounds in Gannan yak milk ghee in the Qinghai–Tibet Plateau was revealed to provide a reference for further key volatile flavor compounds and the formation mechanism of flavor compounds.
